# Sex differences in pre-surgical respiratory physiotherapy impact on hospital and ICU stay in cardiac surgery patients: An observational study

**DOI:** 10.1371/journal.pone.0324207

**Published:** 2025-06-05

**Authors:** Jorge Montero-Cámara, Francisco José Ferrer-Sargues, David Cuesta Peredo, Adrián Sarria Cabello, María José Segrera Rovira, Juan Antonio Margarit Calabuig, Noemí Valtueña-Gimeno, Juan Pardo, María Luz Sánchez-Sánchez

**Affiliations:** 1 Department of Nursing and Physiotherapy, Universidad Cardenal Herrera-CEU, CEU Universities, Alfara del Patriarca, Valencia, Spain; 2 Hospital Universitario de la Ribera, Corbera, Alzira, Valencia, Spain; 3 Department of Mathematics, Physics, and Technology. Universidad Cardenal Herrera-CEU, CEU Universities, Alfara del Patriarca, Valencia, Spain; 4 Physiotherapy in Motion. Multispeciality Research Group (PTinMOTION), Department of Physiotherapy, University of Valencia, Valencia, Spain; NIHR Leicester Biomedical Research Centre, UNITED KINGDOM OF GREAT BRITAIN AND NORTHERN IRELAND

## Abstract

**Introduction:**

Cardiovascular diseases may be amenable to surgical intervention. To mitigate post-surgical complications, diverse strategies are employed, including pre-habilitation programs. This study examines the effect of an unsupervised pre-surgical respiratory physiotherapy program on both sexes in terms of hospital and intensive care unit (ICU) stay lengths, as well as the incidence of post-surgical complications, their severity, and mortality risk.

**Methodology:**

Retrospective observational study of 418 adults who underwent open-heart surgery between 2018 and 2022. The subjects were divided into two sex-based groups based on attendance at individual pre-surgical physiotherapy sessions. A Mann-Whitney U test was employed to evaluate the impact of the pre-surgical respiratory physiotherapy program and a Kruskal-Wallis rank-sum test to assess its influence on both sexes. Additionally, a multiple linear regression analysis was conducted to evaluate the impact of various variables on overall length of stay.

**Results:**

The mean age of women was higher (p = 0.002), and they exhibited longer mean lengths of ICU (p = 0.004) and hospital stays (p = 0.031). In both sexes, a statistically significant reduction in LOS was observed among those undergoing a respiratory physiotherapy program. The linear regression analysis indicated that male sex was associated with a reduction in hospital and ICU stay lengths (p < 0.001). Although women experienced a higher number of complications (p = 0.043), no differences in severity levels or mortality risk were observed between sexes.

**Conclusion:**

An unsupervised home pre-surgical physiotherapy program based on ventilatory exercises can reduce hospital and ICU stay for both men and women. Notwithstanding the higher incidence of complications in women, no differences in severity or mortality risk were observed between sexes.

## Introduction

Cardiovascular disease is the leading cause of death and disability worldwide [1–3]. Its etiology is multifactorial, encompassing metabolic, environmental, behavioral, and social factors [1,2]. These risk factors can be identified systematically or incidentally during routine examinations [4].

When conservative treatment for cardiovascular disease fails, surgery may be necessary [5]. This has enhanced survival in coronary disease patients [6], with coronary artery bypass grafts being the most common [7]. However, surgeries for mitral valve, aortic aneurysms, and atrial fibrillations have also increased significantly [7]. In valvular pathology or when revascularization is required, surgical approaches include percutaneous or thoracotomy [8,9].

The All Patient Refined Diagnostic Related Group (APRDRG) classification is a method for determining procedure costs [10]. The DRG system categorizes patients according to the International Classification of Diseases 10 diagnoses, procedures, age, sex, discharge status, and the presence of complications or comorbidities. In this system, both the level of disease severity and mortality risk associated with the intervention are considered ordinal variables stratified into four grades, calculated based on comorbidities, age, and resource use during admission [11,12].

Hospital and healthcare providers endeavor to minimize the factors that affect the inpatient health status and resource consumption. Thus, intervention protocols reducing the length of anesthesia and intubation are employed, decreasing the hospital length of stay (HLOS), ICU length of stay (ICULOS) and the incidence of hospital-related complications [13,14]. These protocols optimize preoperative conditions for patients through pre-habilitation programs incorporating respiratory and muscular exercise regimens [15,16]. Pre-surgery respiratory exercise programs, focused on optimizing ventilatory physiology and lung inflation, are particularly indicated in thoracic and abdominal surgery to prevent postoperative pulmonary complications, which are the main cause of morbidity and mortality of these patients. These complications include atelectasis, pneumonia, acute respiratory failure and various forms of upper airway obstruction [17–19].

Despite the evidence indicating the efficacy of pre-surgical physiotherapy in cardiac patients scheduled for valve or revascularization surgery [15,16], there is a paucity of studies that have analyzed the differential effects on sex of the pre-surgical physiotherapy outcomes, including inpatients stay, mortality risk, and disease severity stratification according to the APRDRG system.

The aforementioned variables can vary in specific pathologies depending on the sex of the affected individuals [20]. Hormonal fluctuation in the female life cycle, and loss of the cardio-protective action of estrogens may result in an increase in cardiovascular and metabolic risk factors [21–24]. These sex differences may affect the clinical manifestations of the condition, potentially leading to diagnosis and surgical treatment delays [23,25,26]. Evidence suggests that male-based imaging test or clinical presentations may underestimate the severity of valvular or ischemic pathology in women [25–27]. Similarly, sex differences have been identified in the risk of short-term mortality and the incidence of complications following cardiac surgery, with women facing higher risk [28,29].

This study aims to analyze the impact of sex on outcomes after an unsupervised home-based pre-surgical respiratory physiotherapy program, focusing on variables such as the duration of the ICULOS and HLOS, the differences in the percentages of post-surgical and pulmonary complications, as well as the severity and mortality risk overcome by surgery.

## Methodology

The present study is a retrospective observational study that was conducted on a cohort of patients who underwent cardiac surgery via either a thoracotomy or a sternotomy procedure.

The study followed STROBE recommendations for observational studies and complied with the Helsinki Declaration of Ethical Principles for Research Involving Human Subjects. The Hospital de La Ribera Ethical Committee approved the research protocol with code PI 17/122019 and authorized data collection and analysis from patients treated between January 1^st^, 2018 and December 31^st^, 2022, using an anonymized database belonging to the hospital. Ethics Committee waived the requirement for informed consent, due to the nature of the present retrospective study. Access to the patient database was carried out between September and December 2023.

### Study population

The study population included individuals who underwent major surgical procedures between 2018 and 2022. The sample inclusion criteria encompassed adults undergoing valve replacement surgery, bypass, or thoracic/cardiothoracic vessel surgery, irrespective of prior myocardial infarction or whether the surgery was elective or preferential, thus giving priority in the scheduling process. Patients presenting malignant arrhythmia, dementia, or participating in other concurrent studies that could potentially bias the results were excluded.

### Intervention

All patients selected for the study were informed of the necessity and importance of attending an individual pre-surgical physiotherapy session. In this session, the physiotherapist guided the patient through a respiratory home-based exercise program [30,31], enhancing ventilation, coughing capacity, and post-surgery sternal protection. This method enabled unsupervised home exercise. Initially, patients received training on directed ventilation techniques, including various decubitus positions for specific pulmonary area ventilation. In addition, patients were instructed on the correct use of the volumetric incentive spirometer (Portex Coach 2® de 4000ml) and the peak-flow expiration device (Silbemed Datospir Peak-10®). For the spirometer, the maximum inspired volume was calculated per patient, with an exercise protocol of 10 repetitions at 25–30% inspiratory capacity, followed by a 100% inspiration repetition [32,33]. For the expiration device, the maximum volume was calculated per patient in a forced expiratory maneuver. The prescribed protocol was 10 repetitions: three at 25%, 50%, and 75% volume resistance, concluding with one at 100% exhaled volume [32,34].

Then, the exercise session concluded with a review of the recommended sternum protection guidelines following surgery, along with guidance on bed to chair transfers and adapting daily life activities during the weeks following the intervention.

It is recommended that the exercises be performed at least twice a week during the period preceding the surgical procedure. The protocol should be discontinued one day prior to the surgical date.

Finally, the physiotherapist directs the patients to register and subsequently monitor their compliance with the exercise program.

### Outcomes

The descriptive variables included age, sex, concomitant diseases, body mass index, pathology regarding the APRDRG [10,35], surgical type, the Charlson Index, smoking habit, alcohol consumption, and whether the patient participated in the pre-surgical physiotherapy session.

Regarding the independent variables, they were defined as HLOS and ICULOS, quantified in hours. Post-surgical complications, disease severity and mortality risk associated with the surgery were also considered.

### Statistical analysis

Data from hospital records were rigorously reviewed and analyzed using RStudio [36]. Normality and homogeneity of variances were evaluated using Kolmogorov-Smirnov’s and Levene’s tests, and U Mann-Whitney tests identified significant disparities between the study groups.

A significance level of 0.05 was applied to all tests. Pearson’s Chi-squared and Fisher’s exact tests compared differences in percentages between sexes and qualitative factors.

Participants were categorized based on sex and individual pre-surgical physiotherapy session attendance. A Kruskal-Wallis rank sum test with BH p-value adjustment detected significant differences between groups. Pairwise comparisons were presented using the Wilcoxon rank sum test.

Spearman’s correlation and multiple linear regression models identified significant factors influencing the HLOS or ICULOS, as the model’s dependent variables. Covariates included sex, age, postsurgical complications, respiratory status, individual pre-surgical physiotherapy session attendance, substance abuse, severity level, Charlson Index score, and surgery nature. The 95% confidence interval for each regression coefficient was also calculated to measure the effect size.

The “performance” library in R [37] assessed model alignment and calculated quality indices. Posterior predictive checks, homogeneity of variance and normality of residuals tests, and multicollinearity examination were conducted. Influential observations were curtailed using Cook’s distance [38].

A post hoc analysis using the G-Power program [39] determined the final statistical power of the study as 0.99, based on a comparison of two independent means, using a two-tailed test, with a 95% confidence level.

## Results

The study population comprised 418 individuals, including 121 women and 297 men, with a mean and standard deviation (SD) age of 70.4 (10.9) years, a body mass index of 29.8 (4.8), and a Charlson Index of 2.74 (2.0). The intergroup analysis indicated only a statistically significant difference in the mean age between sexes (p = 0.002), showing the women’s group being older. Data are shown in Table 1.

**Table 1:** Descriptive analysis. Mann Whitney comparison between sexes.

Variable	Female n = 121	SEM	Male n = 297	SEM	P-value
Age	72.72 (10.7) 76 [64: 81]	0.973	69.51 (10.8) 71 [64: 77]	0.627	0.002*
BMI	29.30 (5.01) 29 [26: 32]	0.455	29.99 (4.8) 29 [27: 33]	0.278	0.226
Charlson Index	2.57 (1.92) 2 [1: 3]	0.174	2.81 (2.1) 2 [1: 4]	0.122	0.340

n: sample size; P-value: significance; SEM: standard error of the mean. Data expressed as mean (standard deviation) and median [interquartile range].

The mean of the inpatient HLOS was 270.6 hours (251.9) (median (IQR) 187 [145.7; 307.7]), while the mean of the ICULOS was 99.5 hours (161.4) (median (IQR) 52 [41; 96]). In terms of ICULOS, women had a significantly longer duration than men (p = 0.004). As for HLOS, women also showed a significantly longer period than men, which was (p = 0.031).

A total of 192 patients did not attend the individual pre-surgical physiotherapy session and did not follow the program (No pre-surg group), while 226 did (pre-surg group). Regarding the pre-surgical respiratory physiotherapy program effect, home-based exercise resulted in a statistically significant reduction of ICULOS, both in women (p = 0.014) and in men (p = 0.025). Similarly, HLOS also saw statistically significant reductions in both women (p = 0.009) and men (p < 0.001). It is noteworthy that the duration of inpatient stays, whether in the ICU or in general hospital wards, for women who participated in the pre-surgical respiratory physiotherapy program was comparable to that of men who did not participate in the program. Median values and interquartile ranges for each group can be found in Table 2.

**Table 2:** Mann-Whitney comparative analysis between and within sexes in HLOS and ICULOS.

	Female				Male		Diff.	p
ICULOS hours	122.51 (199.01) 57 [45: 113]				90.15 (142.57) 51 [32: 90]		−32.36 [−71.65; 6.93]	**0.004** ^*^
HLOS hours	293.77 (282.04) 196 [151: 334]				261.18 (238.48) 147 [143: 273]		−32.59 [−93.36; 28.18]	**0.031** ^*^
	No pre-surg	Pres-urg	Diff.	p	No pre-surg	Pre-surg	Diff	p
n	48	73			144	153		
ICULOS hours	159.3 (260.5) 86.5 [50; 147.7]	98.3 (142.2) 53 [147; 277]	−60.96 [−139.21; 17.29]	**0.014** ^*^	107.1 (159.3) 54 [41.5; 100.7]	74.2 (123.2) 49 [30; 73]	−32.96 [−65.82; −0.11]	**0.025** ^*^
HLOS hours	361.5 (368.8) 212.5 [166.2; 447]	249.2 (196.8) 190 [147; 277.5]	−112.25 [−225.28; 0.77]	**0.009** ^*^	299.6 (239.7) 205.5 [41.5; 100.7]	224.9 (232.2) 164 [140; 213]	−74.67 [−130.54; −18.79]	**0.001** ^*^

* statistical difference; Diff: means differences with confidence intervals calculated as “male” minus “female”, and “pre-surgical physiotherapy” minus “no pre-surgical physiotherapy”; HLOS: hospital length of stay; ICULOS: intensive care unit length of stay; n: sample size; No pre-surg: no pre-surgical physiotherapy group; P-value: significance; Pre-surg: pre-surgical physiotherapy group. Data expressed as mean (standard deviation) and median [interquartile range]. Kruskal-Wallis test results are expressed using the P-value.

**Fig 1:**
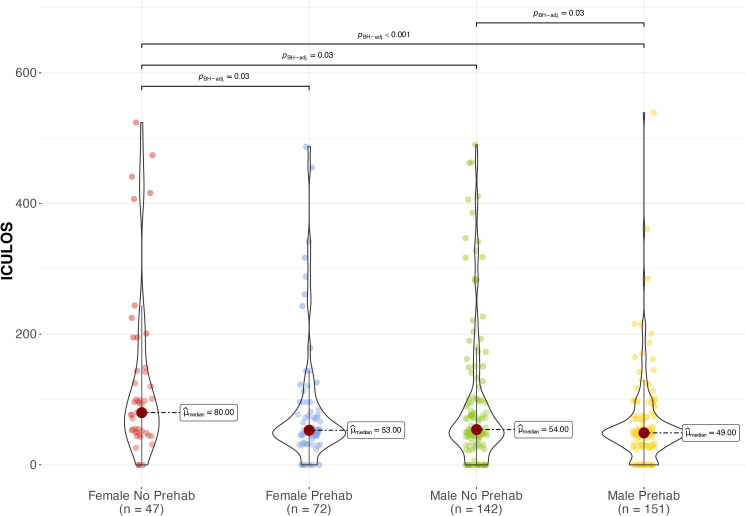
ICULOS comparison between groups by sex and attendance or non-attendance in pre-surgical physiotherapy. **Kruskal-Wallis test.** Female no pre-surg: females who did not attend individual pre-surgical physiotherapy session; Female pre-surg: females who attended individual pre-surgical physiotherapy session; ICULOS: Intensive care unit length of stay in hours; Male no pre-surg: Males who did not attend individual pre-surgical physiotherapy session; Male pre-surg: Males who attended individual pre-surgical physiotherapy session; p: statistical significance value.

**Fig 2:**
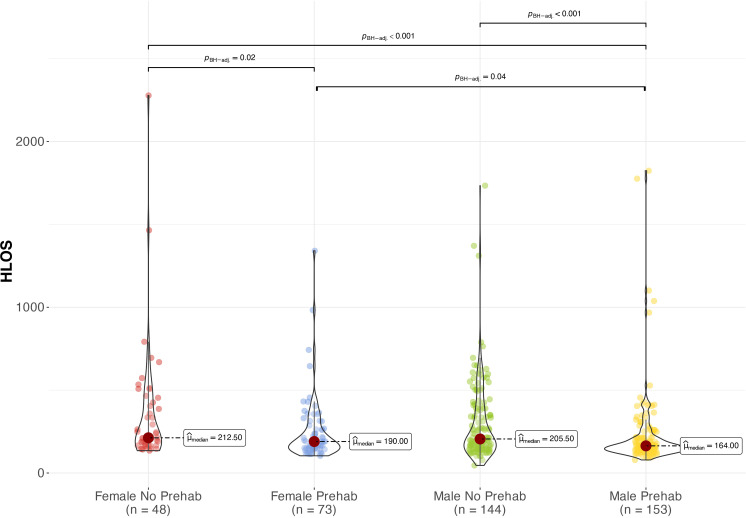
HLOS comparison between groups by sex and attendance or non-attendance in pre-surgical physiotherapy. **Kruskal-Wallis test.** Female no pre-surg: females who did not attend individual pre-surgical physiotherapy session; Female pre-surg: females who attended individual pre-surgical physiotherapy session; HLOS: Hospital length of stay in hours; Male no pre-surg: Males who did not attend individual pre-surgical physiotherapy session; Male pre-surg: Males who attended individual pre-surgical physiotherapy session; p: statistical significance value.

Women who did not participate in the pre-surgical respiratory physiotherapy program had a significantly longer ICULOS of 159.3 hours (260.5) compared to men, who spent only 107.1 hours (159.3) (p = 0.03) (Fig 1). Notwithstanding, in the pre-surgical respiratory physiotherapy program groups, women had a HLOS of 249.2 hours (196.8), while men had a reduced duration of 224.9 hours (232.2), resulting in a statistically significant difference (p = 0.042) (Fig 2).

Regarding the APRDRG, 288 patients underwent surgical intervention for valve pathologies, 110 received coronary revascularization, and 20 were treated for vascular cardiothoracic or thoracic pathologies. Concerning pathology and associated complications, all APRDRGs demonstrate a statistically significant reduction in HLOS. This is also observed in ICULOS, except for APRDRG 165 [35], which pertains to coronary artery bypass grafts with acute myocardial infarction or other diagnostic complications.

The results of the multiple linear regression analysis indicated that male sex was associated with a reduction in the HLOS and ICULOS of approximately 16 hours (p < 0.001). Similarly, the performance of a pre-surgical respiratory physiotherapy program (p < 0.001) resulted in a reduction of 11.08 hours in the duration of ICULOS and 24.95 hours in the duration of HLOS. Moreover, while there is no statistical correlation between age and ICULOS (P = 0.151), a correlation was found between age and HLOS (p = 0.005). All data can be found in Table 3.

**Table 3:** Multiple linear regression.

HLOS						ICULOS					
Variable	b Coeff.	Std. error	T value	P-value	C.I. 95%	Variable	b Coeff.	Std. error	T value	P-value	C.I. 95%
Intercept	204.04	20.86	9.77	<0.001*	[207.13, 341.51]	Intercept	100.93	15.49	6.51	**<0.001** *	[71.14, 137.33]
Age	0.63	0.22	2.82	**0.005** *	[0.11, 1.07]	Sex (male)	−16.06	3.31	−4.83	**<0.001** *	[−24.08, −10.92]
Sex (male)	−16.15	4.70	−3.43	**<0.001** *	[−58.37, −13.03]	Pre-surgical physiotherapy (yes)	−11.08	3.03	−3.65	**<0.001** *	[−17.92, −5.91]
Pre-surgical physiotherapy (Yes)	−24.95	4.41	−5.65	**<0.001** *	[−24.10, −4.01]	Charlson Index	4.86	0.98	4.92	**<0.001** *	[2.96, 6.67]
Charlson Index	5.69	1.27	4.45	**<0.001** *	[2.58, 8.73]	Need for reintervention	145.19	16.17	8.97	**<0.001** *	[94.89, 180.82]
Pneumothorax	81.91	27.43	2.98	**0.003** *	[14.77, 127.82]	Pleural effusion	70.24	7.25	9.68	**<0.001** *	[58.31, 86.67]
Need for reintervention	185.48	15.21	12.19	**<0.001** *	[152.07, 220.55]	Atrial fibrillation	32.70	5.37	6.08	**<0.001** *	[20.86,41.92]
Pleural effusion	146.13	16.98	8.60	**<0.001** *	[106.44, 167.80]	Respiratory insufficiency	53.49	21.90	2.44	**0.015** *	[10.57, 96.41]
Atrial fibrillation	62.94	8.66	7.26	**<0.001** *	[54.86, 90.48]	Fever	48.62	13.03	3.72	**<0.001** *	[24.99, 75.42]
Pericardial effusion	73.68	19.54	3.77	**<0.001** *	[31.95, 98.50]	Maintained arterial hypotension	−49.43	15.39	−3.21	**0.001** *	[−78.64, −19.09]
Other post-surgical complications	84.45	14.00	6.03	**<0.001** *	[29.85, 82.66]	Severe severity	15.92	5.38	2.95	**0.003** *	[5.82, 26.90]
Arterial hypertension	−12.70	0.05	2.51	**0.01** *	[−25.75, −4.64]	Extreme severity	281.61	22.31	12.62	**<0.001** *	[224.06, 343.09]
Severe severity	69.65	10.12	6.87	**<0.001** *	[47.08, 85.13]	APRDRG 163	−36.50	11.33	−3.22	**0.001** *	[−58.42, 5.57]
APRDRG 163	−73.22	16.65	−4.39	**<0.001** *	[−105.64, −35.64]	APRDRG 166	−41.05	11.52	−3.56	**<0.001** *	[−67.06, −15.42]
APRDRG 165	−44.02	21.82	−2.02	**0.045** *	[−135.28, −25.16]	APRDRG 167	−36.33	14.24	−2.55	**0.011** *	[−73.06, −10.75]
APRDRG 166	−78.67	16.89	−4.65	**<0.001** *	[−114.56, −43.64]						
APRDRG 167	−70.01	21.79	−3.12	**<0.001** *	[−120.36, −37.81]						

APRDRG 163: procedures on valves without acute myocardial infarction or complex diagnosis; APRDRG 165: coronary bypass with acute myocardial infarction or complex diagnosis; APRDRG 166: coronary bypass without acute myocardial infarction or complex diagnosis; APRDRG 167: other cardiothoracic and thoracic vascular procedures; b Coeff: Betta coefficient; HLOS: hospital length of stay; ICULOS: intensive care unit length of stay; P-value: significance; std Error: standard error.

To conclude, we would like to remark on the relationship between sex and the different APR-DRGs. DRG 163 is more common in men (p = 0.0007). The proportion of individuals of each sex for each APR-DRG can be found in the Supporting Information material (S1 Table).

The analyzed population exhibited a prevalence of arterial hypertension, dyslipidaemia and Type II Diabetes Mellitus, respectively, at 70.3%, 45.2% and 35.2%. At the time of the individual pre-surgical physiotherapy session, 17% of individuals acknowledged being smokers, and 21.3% admitted to having consumed alcohol in the preceding days. Of individuals scheduled for surgery, 54.1% attended the individual pre-surgical physiotherapy session. Post-surgery, 41.1% suffered from complications associated with the procedure, with respiratory complications accounting for 9.6% of these cases.

Despite the statistically higher number of men reporting alcohol consumption and cigarette smoking compared to women (p < 0.001), the prevalence of arterial hypertension, dyslipidemia, and diabetes mellitus showed no significant differences between the two groups. No significant differences were observed between sexes in the number of patients who attended the individual pre-surgical physiotherapy session.

However, 48.8% of women suffered some type of postoperative complication. On the other hand, only 38% of men experienced complications, indicating a statistically significant reduction (p = 0.043). In the percentage of postoperative complications of a pulmonary nature, women suffered more pulmonary complications than men (p = 0.006). Information on the frequencies of these variables can be found in Table 4.

**Table 4:** Frequency descriptive analysis.

Variable	Female		Male		P-value
	n	Percentage	n	Percentage	
Complication	59	48.8%	113	38%	0.043*
Respiratory complication	19	15.7%	21	7.1%	0.006*
Diabetes mellitus type 2	36	29.8%	111	37.4%	0.138
Hypertension	86	71.1%	208	70%	0.832
Dyslipidaemia	54	44.6%	135	45.5%	0.877
Pre-surgical physiotherapy (Yes)	73	60.3%	153	51.5%	0.101
Smoke	14	11.6%	57	19.2%	<0.001*
Alcohol	5	4.13%	84	28.28%	<0.001*
Planned	112	92.6%	262	88.2%	0.189
Severity ind. low	24	19.8%	74	24.9%	0.709
Severity ind.moderate	62	51.2%	142	47.8%	
Severity severe	28	23.1%	62	20.9%	
Severity ind.extreme	28	5.79%	19	6.4%	
Mort. Risk low	32	26.4%	109	36.7%	0.202
Mort. Risk moderate	61	50.4%	121	40.7%	
Mort. Risk severe	18	14.9%	45	15.2%	
Mort.Risk Extreme	10	8.3%	22	7.4%	

Chi-square, Mort. Risk: mortality risk; n: sample size; p-value: significance; Severity ind: severity index. Data expressed as mean (standard deviation) and median [interquartile range].

## Discussion

The aim of the present study is to find out whether a home-based, unsupervised pre-surgical respiratory physiotherapy program can have any effect on patients in relation to sex. The data show that even if the patients who complete the pre-surgical rehabilitation program experience a decrease in the HLOS and ICULOS, women tend to remain longer in both situations, even for more time than men who do not comply with the pre-surgical rehabilitation program. This and the greater number of postoperative complications in women could be explained by the higher average age of the female group compared to men. However, the multiple regression shows that there is only a correlation between age and length of stay, and only with an increase of 36 minutes per additional year of life. Such a minor difference does not seem to explain the discrepancies between the sex groups, when the difference in mean age is three years and the median is of five years. Higher age and higher percentage of complications in the female group can be explained, as other studies suggest, by the later detection of risk factors compared to the male population. This makes implementing a prevention program for cardiovascular disease before it manifests itself unlikely [40–42]. In addition, the images may underestimate the severity of the cardiovascular pathology in the female population [25,27], and lead to a delayed diagnosis, and, therefore, to a premature surgery [23,25,26]. Also, women tend to suffer from more comorbidities than men [43–45]. However, in the present study, no statistically significant differences in severity or Charlson Index were found between the sexes.

The pre-surgical respiratory physiotherapy program had remarkable effects in both groups; both sexes reduced the ICULOS by approximately 33 hours for patients who completed the program, while the reduction in ICULOS for the female group increased to 122 hours compared to 74 hours for the male group. This reduction in length of stay may be due to hemodynamic improvement resulting from the use of the volumetric incentive spirometer [46,47]. Although each patient performed the exercises after surgery, the patients performing the pre-surgical respiratory physiotherapy program facilitated regaining respiratory muscle strength [48] and thus improved ventilatory parameters [49,50].

This study found no differences in mortality risk between the sexes, but other studies have associated women with a higher risk of death in the next 30 days after open surgery [28,29,43–45]. Neither difference was found between the sexes about the severity of illness, consistent with the results presented in other studies [28].

### Study limitations

This study acknowledges certain limitations, for instance, the gender disparity in the participant pool. However, it is noteworthy that the observed lower female participation aligns with the figures reported in the Clinical Indicators Report of the Spanish Health Ministry [51] and those published by the European Society of Cardiology [2], as well as global epidemiological studies [52,53].

Also, there was no follow-up of individuals who attended the pre-habilitation session made directly by the hospital staff, so it cannot be known with certainty whether they adhered fully to the program, even if patients were instructed to remain accountable and follow their progress. It was necessary to rely on patients’ commitment to the exercises.

It is acknowledged that a home-based program may not be without flaws in terms of monitoring but may also be beneficial for patients who are unable to participate in pre-habilitation in hospital settings. This approach has the potential to alleviate pressure on hospital services, thereby providing an additional option for those who would otherwise be unable to access pre-habilitation.

Finally, the present study was conducted at a single medical center. This approach prevents direct comparison with other studies conducted in multiple centers. However, in terms of sample size, this design allows for the inclusion of a larger patient cohort.

It is recommended that future research prioritize the use of randomized controlled trials to investigate the effects of a monitored home-based, unsupervised pre-surgical respiratory physiotherapy program. The objective should be to evaluate the efficacy of the program in comparison to other programs in terms of complications, HLOS and ICULOS, with a particular emphasis on discerning potential sex differences.

## Conclusion

An unsupervised home-based preoperative respiratory physiotherapy program appears to achieve a similar reduction in the ICULOS for both men and women following thoracic surgery. Despite the female group being older at the time of surgery, having more post-surgical complications, and spending longer in the hospital and in the ICU than the male group, the study found no significant differences in mortality risk associated with the surgery between the sexes.

## Supporting information

S1 Table Percentage of men and women in each APR-DRG. (DOCX)
