# Supplementary material for: Sex differences in pre-surgical respiratory physiotherapy impact on hospital and ICU stay in cardiac surgery patients: An observational study
Source: PLoS One. 2025 Jun 5;20(6):e0324207. doi: 10.1371/journal.pone.0324207 (PMC12140195; doi:10.1371/journal.pone.0324207)
Supplement: S1 Table — (DOCX) [file pone.0324207.s001.docx]

**S1 Table. Percentage of men and women in each APR-DRG.**

| APRDRG | Female | Male | Total | P-value |
| --- | --- | --- | --- | --- |
| 162 | 5 (4.13%) | 11 (3.70%) | 16 (3.83%) | 0.0007* |
| 163 | 95 (78.51%) | 177 (59.60%) | 272 (65.07%) |  |
| 165 | 2 (1.65%) | 19 (6.40%) | 21 (5.02%) |  |
| 166 | 13 (10.74%) | 76 (25.59%) | 89 (21.29%) |  |
| 167 | 6 (4.96%) | 14 (4.71%) | 20 (4.78%) |  |

162: procedures on valves with acute myocardial infarction or complex diagnosis, 163: procedures on valves without acute myocardial infarction or complex diagnosis, 165: coronary bypass with acute myocardial infarction or complex diagnosis, 166: coronary bypass without acute myocardial infarction or complex diagnosis, 167: other cardiothoracic and thoracic vascular procedures, *Fisher’s exact test.
